# Boolean model of the gene regulatory network of *Pseudomonas aeruginosa* CCBH4851

**DOI:** 10.3389/fmicb.2023.1274740

**Published:** 2023-11-30

**Authors:** Márcia da Silva Chagas, Marcelo Trindade dos Santos, Marcio Argollo de Menezes, Fabricio Alves Barbosa da Silva

**Affiliations:** ^1^Scientific Computing Program (PROCC), FIOCRUZ, Rio de Janeiro, Brazil; ^2^National Laboratory of Scientific Computing, Rio de Janeiro, Brazil; ^3^Institute of Physics, Fluminense Federal University, Niterói, Brazil

**Keywords:** Boolean model, gene regulatory network (GRN), pseudomonas aeruginosa, system biology and systems modeling, multidrug resistance (MDR)

## Abstract

**Introduction:**

*Pseudomonas aeruginosa* infections are one of the leading causes of death in immunocompromised patients with cystic fibrosis, diabetes, and lung diseases such as pneumonia and bronchiectasis. Furthermore, *P. aeruginosa* is one of the main multidrug-resistant bacteria responsible for nosocomial infections worldwide, including the multidrug-resistant CCBH4851 strain isolated in Brazil.

**Methods:**

One way to analyze their dynamic cellular behavior is through computational modeling of the gene regulatory network, which represents interactions between regulatory genes and their targets. For this purpose, Boolean models are important predictive tools to analyze these interactions. They are one of the most commonly used methods for studying complex dynamic behavior in biological systems.

**Results and discussion:**

Therefore, this research consists of building a Boolean model of the gene regulatory network of *P. aeruginosa* CCBH4851 using data from RNA-seq experiments. Next, the basins of attraction are estimated, as these regions and the transitions between them can help identify the attractors, representing long-term behavior in the Boolean model. The essential genes of the basins were associated with the phenotypes of the bacteria for two conditions: biofilm formation and polymyxin B treatment. Overall, the Boolean model and the analysis method proposed in this work can identify promising control actions and indicate potential therapeutic targets, which can help pinpoint new drugs and intervention strategies.

## 1 Introduction

Some of the life-threatening nosocomial infections among severely ill and immunocompromised individuals are bacterial infections caused by the “ESKAPE” pathogens (Rice, 2010). This is an acronym for *Enterococcus faecium*, *Staphylococcus aureus*, *Klebsiella pneumoniae*, *Acinetobacter baumannii*, *Pseudomonas aeruginosa*, and *Enterobacter* spp., which are characterized by their ability to escape the action of multiple drugs (Santajit and Indrawattana, 2016). Consequently, *P. aeruginosa* and some of these multidrug-resistant (MDR) pathogens have been classified by the World Health Organization as Priority 1, the most critical group on the list of pathogens for research and development of new antibiotics (World Health Organization [WHO], 2017).

Multidrug resistance is the central challenge in selecting appropriate antibiotic treatments and reduces treatment options, especially in nosocomial settings such as healthcare-associated infections (HAIs) (Matos et al., 2018; Tamma et al., 2021), which pose a serious public health problem due to high rates of morbidity and mortality in hospitalized patients and high healthcare costs, with *P. aeruginosa* being one of the most prevalent agents (Litwin et al., 2021).

*Pseudomonas aeruginosa* is a ubiquitous opportunistic pathogen that can cause infections in the lower respiratory tract, skin, urinary tract, eyes, soft tissues, surgical wounds, and gastrointestinal system, among others, leading to bacteremia, endocarditis, and other complications, mainly in hospital settings and immunocompromised patients (Horcajada et al., 2019; Medeiros Filho et al., 2019; Montero et al., 2020). It is the leading cause of death in patients with cystic fibrosis, diabetes, and other lung diseases such as pneumonia and bronchiectasis, but rarely causes infection in immunologically healthy individuals (Vallet-Gely and Boccard, 2013). This non-fermenting gram-negative bacterium is one of the most challenging to treat (Kadri et al., 2018) due to its intrinsic resistance, acquisition of resistance through chromosomal genetic mutations, and horizontally acquired mechanisms of multidrug resistance (Horcajada et al., 2019). Indeed, patients infected with MDR clones of *P. aeruginosa* have a higher mortality rate (44.6%) than those infected with non-MDR strains (24.8%) (Matos et al., 2018).

The production of carbapenemases is the most epidemiologically important mechanism of carbapenem resistance. Among clinical MDR isolates of *P. aeruginosa* in Brazil, São Paulo metallo-β-lactamase (SPM-1) is the most prevalent carbapenemase (Martins et al., 2018). This enzyme is encoded by the blaSPM-1 gene, located on the chromosome of *P. aeruginosa* (Medeiros Filho et al., 2019), conferring resistance to almost all classes of beta-lactams. The first reported strain of MDR *P. aeruginosa* carrying the blaSPM-1 gene in Brazil dates back to 2003 (Gales et al., 2003). SPM-1-producing *P. aeruginosa* is associated with the SP/ST277 clone and has been isolated from hospital sewage, rivers, and migratory bird microbiota, being widely disseminated in various Brazilian geographic regions (Nascimento et al., 2016; Martins et al., 2018). The strain *P. aeruginosa* CCBH4851, which this research work is based on, belongs to the SP/ST277 clone and was involved in an endemic outbreak in Brazil in 2008, being isolated from the tip of a catheter in a hospitalized patient (Silveira et al., 2014). This strain is resistant to most clinically important antimicrobials, being susceptible only to polymyxin B, and possesses various mechanisms of mobile genetic elements (Silveira et al., 2014; Medeiros Filho et al., 2019).

A more comprehensive understanding of *P. aeruginosa* behavior can be obtained by analyzing the dynamics of its gene regulatory network (GRN) due to the predicted gene expression patterns (Chagas et al., 2022). In recent years, computational modeling methods have been employed to simulate complex biological processes influenced by numerous factors, including the construction of biological networks and analysis of gene, metabolic, signal transduction pathways, and/or protein interactions (Das et al., 2010; Tatarinova and Nikolsky, 2017). The GRN consists of molecular regulators (RG, regulatory genes), including transcription factors, that interact with each other and other molecules within the cell to regulate mRNA levels and protein expression (Souza, 2015). The GRN is modeled as a network, where vertices represent genes, and the connections represent two types of interactions: gene expression activation or inhibition (Garg et al., 2011).

Boolean networks are a particularly simple way to model a complex system (Albert et al., 2008). In a Boolean model of the GRN, the nodes represent genes, the edges represent activation or repression interactions between them, and each node can be in an ON state (1, meaning expressed) or an OFF state (0, meaning not expressed) (Schwab et al., 2020). In a simulation, each gene’s expression level (state) is functionally related to the expression states of certain other genes, depending on its logical updating rule (Banzhaf et al., 2020).

Among the modes of state transition from state x(t) to its successor state x(t + 1) in Boolean networks, three are more frequently used: synchronous, asynchronous, and probabilistic (Schwab et al., 2020). All updating methods can lead to stable and significant behaviors of biological dynamics (Wang et al., 2012). In the probabilistic mode, one function per node is randomly chosen at each time step according to its probability before each state transition, and then synchronous updating is performed. The probabilistic Boolean network (PBN) mode was proposed by Shmulevich et al. (2002) to relax the deterministic rigidity of the Boolean model and incorporate uncertainty in gene expression data. In all types of Boolean networks, including PBNs, the dynamics of the model can be represented in state transition graphs, which show the transition between states of all nodes in the network and their progression toward each attractor (Schwab et al., 2020).

**FIGURE 1:**
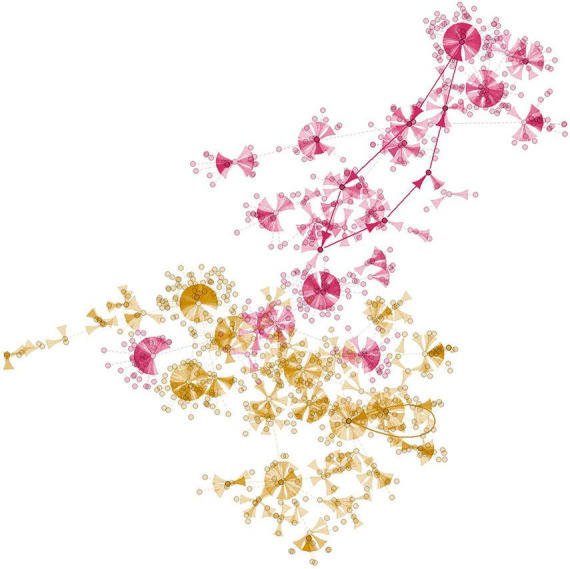
An example of basins of attraction state graph from literature. These basins are from the mammalian cell cycle network introduced by Fauré et al. (2006) and developed by Müssel et al. (2023). This figure was generated by us. There are 10 genes and 1024 states, leading to two basins of attraction, each comprising 512 states. The nodes are specific network states, and each arrow is a state transition, with attractors highlighted by bold lines. The pink basin of attraction corresponds to attractor 1 and the yellow basin to attractor 2. We used the BoolNet Package (Müssel et al., 2023) to generate this figure.

Attractors are sequences of states that repeat periodically, representing long-term behaviors of Boolean networks and being associated with biological phenotypes, making them a crucial point of interest in model analyses (Wang et al., 2012). They resemble what is observed in cells, which are stable systems where their behavior (expression pattern) does not change unless there are modifications in environmental conditions or genetic mutations (Ma’ayan, 2012). In other words, cells operate within attractors of the system, representing specific cellular behaviors (Naldi et al., 2015). The compilation of all states leading to an attractor is called the basin of attraction. Therefore, the larger the basin of attraction is, the higher the probability that the attractor is biologically significant (Schwab et al., 2020). Generally, the attractor with the largest basin of attraction describes a well-known system behavior as it represents the most likely cellular behavior (Klemm and Bornholdt, 2005). Waddington’s “epigenetic landscape” concept (Waddington, 1957) can represent the global dynamics of a GRN by formally modeling cellular functioning through attractor theory (Huang et al., 2009). The epigenetic landscape is a space of states that defines the connection between an organism’s genotype and phenotype; altering it can lead to different phenotypes. Hypothetically, it is possible to manipulate essential genes within the basins of attraction to alter the expected phenotype. The description of attractors and their basins of attraction is based on a set of gene states within the analyzed model. Attractors do not contain genes; instead, they are linked to specific states. A particular gene may be active or inactive, occurring in multiple basins of attraction across different phenotypes without forming a direct gene-attractor association. For example, in Figure 1, we see an example of attractor basins within the mammalian cell cycle network, as introduced by Fauré et al. (2006) and discussed by Müssel et al. (2023). There are 10 genes and 1024 states, resulting in two basins of attraction with 512 states each. Each node within the state graph of the mammalian cell cycle network represents a specific network state, and each arrow represents a state transition. Different basins of attraction are distinguished by varying colors, with the yellow basin corresponding to attractor 1 (bold lines in yellow) and the pink basin corresponding to attractor 2 (bold lines in pink).

According to Sgariglia et al. (2021), biologically relevant attractors and basins of attraction can be identified through trajectory simulations, where the initial points are binary gene expression values obtained by binarized cellular data, such as RNA-seq data. The essential genes contributing to the stability of the resulting basins of attraction can be considered potential therapeutic targets as they can modify the epigenetic landscape in which they are involved.

The state space of Boolean networks grows exponentially with the number of nodes. In this work, due to the size and complexity of the GRN of *P. aeruginosa* CCBH 4851 (Chagas et al., 2022), there was a need to reduce the GRN into a core sub-network. Kauffman et al. (2003) describe that the definition of the core sub-network for attractor calculation should consider all nodes with at least one outgoing edge, which can change the network state from one timestep t to the next. Therefore, for this work, the core sub-network should consider all regulatory genes, and only them Regulatory genes can be of two types: Regulators only, with at least one outgoing edge, and regulator/target, with at least an outgoing edge and at least one incoming edge, being also targets of other regulators.

In a Boolean model, functions can be random, biologically derived, or follow an order, such as Kauffman’s canalizing functions (1974) (Kauffman, 1974). A canalizing function, as per Kauffman’s definitions, takes multiple input variables, typically representing gene states, and generates an output (0 or 1, “off” or “on” state). These functions have one or more “dominant” input variables, which can dictate the function’s output, regardless of the states of other input variables. When one of these dominant inputs is in a specific state, it “canalizes” or locks the function’s output, making it independent of the other input values. The non-dominant inputs can influence the function’s outcome only if the dominant inputs are in particular states. In the absence of dominant inputs, the secondary inputs alone can determine the function’s output. Their role in GRN models captures the dynamics and stability of biological systems, and over the years, more evidence has emerged that canalization is ubiquitous in gene regulation (Daniels et al., 2018). In more than 130 curated GRN models, the majority of regulatory rules used are called Nested Canalizing Functions (Kadelka et al., 2020), which has increased interest in studying these Boolean behaviors and their impact on GRN dynamics and controllability (Murrugarra and Dimitrova, 2015). It has been observed that Boolean networks governed by canalizing functions are typically more stable than those governed by random functions (Kauffman et al., 2003), as they have a smaller number of attractors and are more robust to perturbations (Dimitrova et al., 2022). In general, the greater the quantity and prevalence of canalization, the more stable the dynamics of the model (Karlsson and Hörnquist, 2007).

Canalizing Boolean functions is suitable for Boolean network models of GRNs, as they represent biological regulations well (Harris et al., 2002; Nikolajewa et al., 2007). Nested Canalizing Functions (NCFs), a subclass of canalizing functions, were more recently introduced (Harris et al., 2002) and studied from the perspective of the stability properties of network dynamics. NCFs exhibit an even more specific canalizing behavior due to their “nested” hierarchical canalization. Within an NCF, you can find multiple layers of canalization. In this structure, the state of one input variable determines the canalization of another input variable, and this pattern can continue through multiple layers. This nested characteristic of canalization introduces an additional layer of complexity to the function. Most regulatory rules in molecular regulatory networks are canalizing, most of which are nested canalizing (Kauffman et al., 2004; Murrugarra and Laubenbacher, 2011). Therefore, they are important given their relevance in systems biology. An important characteristic of NCFs, according to Li et al. (2013), is that they exhibit a stabilizing effect on the dynamics of a Boolean network: small perturbations from an initial state do not increase over time and eventually end up in the same attractor as the initial state.

In this work, we propose a Boolean model of the *P. aeruginosa* CCBH4851 GRN based on Nested Canalizing Functions and study its dynamics. For attractor landscape characterization, we used RNA-seq data generated under several conditions. We then discuss attractors and genes related to specific experiments, such as biofilm formation and Polymyxin B treatment. The Boolean model and analysis presented in this work provide important directions on the dynamic behavior of *P. aeruginosa* and can lead to new intervention strategies for multidrug-resistant strains.

## 2 Materials and methods

**FIGURE 2:**
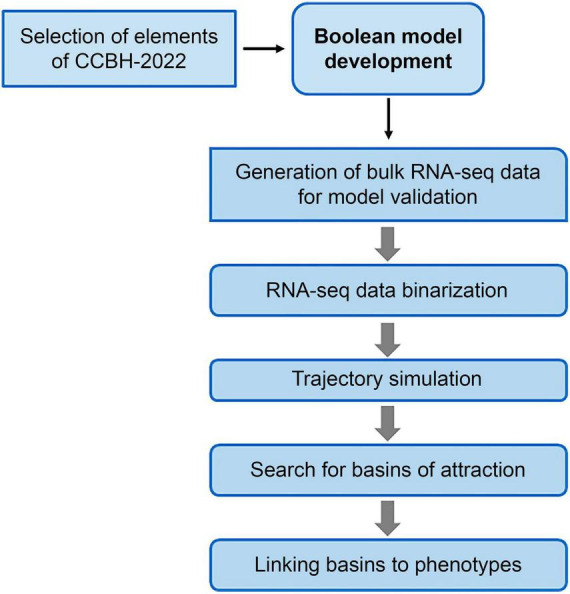
Flowchart of the steps for constructing the Boolean model of the RRG of Pseudomonas aeruginosa CCBH4851. Due to the size and complexity of CCBH-2022, it was necessary to reduce its size to a core sub-network. After determining the nodes that will be included in this core sub-network, the Boolean model is constructed. To analyze its dynamics and identify the attractor basins, bulk RNA-seq data were binarized and used as the initial point for simulating the trajectory of the model. Finally, genes in the basins were associated with bacterial phenotypes through bibliographic research.

The main stages of the method used in this work are illustrated in Figure 2. The following subsections detail the procedures used in those stages.

### 2.1 Boolean model construction

The CCBH-2022 GRN (Chagas et al., 2022) is the largest and most comprehensive version of a *P. aeruginosa* GRN published to date, with edges classified as activation, repression, dual, and unknown, as described in dedicated biological databases and scientific literature. The Boolean model of CCBH-2022 proposed in this work is a PBN due to the uncertainty of dual and unknown edges, with uniform probability values (*P* = 0.5) for expressed function (Boolean value 1) and non-expressed function (Boolean value 0) (Shmulevich et al., 2002).

CCBH-2022 consists of 5,428 regulatory interactions among 3,139 gene products, of which 212 were identified as regulatory genes and 2,927 as target genes. It represents approximately 48% of the *P. aeruginosa* CCBH4851 genome and includes 3,821 positive regulation interactions, 643 negative regulation interactions, 19 double regulation interactions, and 945 unknown interactions (Chagas et al., 2022).

Due to the original network’s complexity and size, a core sub-network was selected to simplify the model, define biologically relevant attractors, and construct the PBN. Thus, the core sub-network consists of 166 genes with both incoming and outgoing edges and 46 genes with only outgoing edges, totaling 212 genes. These 46 nodes always have conserved initial states, as it is through the incoming edge that the state of a gene can be modified. The initial conditions of the core sub-network (which in this work are the binarized bulk RNA-seq expression data) can be defined as a starting point for the trajectory simulation, but the landscape of attractors does not depend on the initial conditions (Kauffman et al., 2003).

The adopted Boolean function assignment for all nodes in the network is the Nested Canalizing Functions (NCFs). As an illustration, in the expression (*ihf* | *rpoD*) and (! (*psrA*)), if *ihf* or *rpoD* = 1, the total resulting expression value will be 1 only if *psrA* is not active. The first group of variables will be equal to 1 only if *psrA* equals 0. In other words, several variables simultaneously influence the value of the total function, with *psrA* dominating the others.

As there is a need to model the dynamics of a large-scale PBN, the selected computational tool was the Approximate Steady-State Analyzer of Probabilistic Boolean Networks (ASSA-PBN), which allows for the analysis of PBNs with a high number of nodes and was presented by Mizera et al. (2018). It simulates state transitions and computes the probability associated with a set of states under analysis.

To generate the input file for ASSA-PBN, which is the Boolean model itself, a Python script was developed by our research group, available in the supplementary data repository. It reads the CCBH-2022 CSV file, selects the core sub-network (genes with at least one outgoing edge), separates the conserved genes, and generates a file with their Boolean rules. This file is in the “.pbn” format, which ASSA-PBN requires.

The next step is assigning the corresponding bulk RNA-seq expression values to each element of the CCBH-2022 core sub-network.

### 2.2 RNA-seq experiments

RNA-seq data are available in the supplementary data repository. The RNA-seq experiments were performed with 16 samples under 8 conditions:

1. Succinate (experiments 1 and 2).
2. Acetate (3 and 4).
3. Glycerol (5 and 6).
4. Glucose (7 and 8).
5. Planktonic (9 and 10).
6. Biofilm (11 and 12).
7. Polymyxin B (13 and 14).
8. Imipenem (15 and 16).

Several phenotypes of *P. aeruginosa* PAO1, among other strains, with their respective regulatory genes, are described in the literature. After constructing the Boolean model of CCBH-2022, trajectory simulations were performed using the RNA-seq data as initial points, and the basins of attraction for each RNA-seq sample were obtained. A search was then conducted in the basins associated with the above-mentioned phenotypes to observe the state of the regulatory genes described in the scientific literature. If the literature shows that certain genes act as positive or negative regulators of a phenotype, we verify whether they are expressed in the related basins. We conducted a search for publications that provide descriptions of the phenotypes of *P. aeruginosa* in the context of the RNA-seq experiments, along with information about the regulatory genes associated with these conditions. These publications may identify genes that have either a positive or negative influence on specific phenotypes. For instance, a gene might be described as directly promoting (i.e., activating) the expression of another gene well-known for its role in biofilm formation or as suppressing (inhibiting) a gene widely recognized for its contributions to antibiotic resistance or efflux pumps, and so forth.

The biofilm and polymyxin phenotypes were selected to validate our Boolean model due to the information available in the literature.

### 2.3 RNA-seq bioinformatics analysis

The bioinformatics analysis for RNA-seq began with quality control of the raw sequences (in fastq format), alignment to a reference genome or transcriptome, quantification, and normalization (steps performed by members of our research group).

Quality control of the raw sequences obtained from an Illumina MiSeq sequencer was performed using FastQC software (Andrews et al., 2016), which allows for observing read quality, nucleotide distribution, GC content, overrepresented sequences, k-mer frequency, and duplication level, among other. With this information, the state of the reads is identified, and measures are taken to optimize them. No sequences were trimmed for this study.

Bowtie2 (Langmead et al., 2019) was used to align the reads, an ultrafast and memory-efficient tool for aligning sequencing reads to long reference sequences. The NCBI reference sequence CP021380.2 was used for this process.

The resulting raw data were normalized to generate a set of values proportional to expression levels (Kauffman et al., 2003). The Feature Aggregate Depth Utility (FADU) tool (Chung et al., 2021) was used for this process. It is a quantification tool specifically designed for prokaryotic transcriptomic analyses, addressing the deficiencies centered around this step. From an alignment file generated by read alignment to a complete genome set, FADU handles ambiguous multigene fragments, assigning fragment counts proportionally (Chung et al., 2021). The FADU workflow uses BAM (Binary Alignment Map) and GFF (General Feature Format) annotation files to identify proportional read counts for prokaryotic RNA-Seq analyses.

The BAM file obtained from Bowtie and a GFF annotation file of the used reference sequence (CP021380.2.gff) were used in FADU to identify proportional read counts for prokaryotic RNA-Seq analyses. A gene count file normalized by TPM was then generated.

The expression data were organized in table form and saved in CSV files, with the names identifying the processed genes. After normalization, the next necessary operation for modeling the dynamics of the core sub-network is binarizing the assigned gene expression values for each unique node using a separation threshold determined by the binarization algorithm. We used the Binarize package available in the R language. Studies show comparative results between BASC-A and BASC-B and conclude that BASC-B performs better in binarized gene expression data (Hopfensitz et al., 2012), which was chosen for this project (*p* < 0.001).

We also used *p*-value validation criteria, and the chosen criterion for this work was the false discovery rate (FDR), which is considered an important metric for assessing the overall confidence of datasets in various biological research areas (Benjamini et al., 2001; Reiner et al., 2003; Glickman et al., 2014). The binarization script applied at this stage is written in the R programming language and is available in the supplementary data repository.

### 2.4 RNA-seq data binarization

Binarization is a strategy that aims to convert continuous gene expression profiles into binary information (Müssel et al., 2016). In this work, we perform a transformation of the input data (real numbers) into discrete values (binaries), as we are modeling a real phenomenon (gene expression values) using a Boolean model (Zhao et al., 2021). Since the data are bulk RNA-seq data, the values of each gene were divided into two clusters, with similar values grouped together (partitions). A threshold value x is defined within the range of extremes in each sample, where values greater than x are classified into one cluster, while values lower than x are classified into the other (Müssel et al., 2016).

Specialized algorithms, such as BASC-A and BASC-B (Binarization Across multiple SCales) (Hopfensitz et al., 2012), are used in the binarization process based on the volume of processed data. These algorithms analyze whether the variation in the expression of real numbers for each gene justifies partitioning (Reis, 2013). These algorithms use the ordered vector of input values and evaluate them through successive approximations until finding the boundary between the two clusters, identifying the “best” threshold for binarization based on the data itself (Ferreira, 2019).

The BASC method is ideal for analyses involving the reconstruction of Boolean networks, especially when conventional methods result in multiple solutions, and it is also useful in reconstructing PBNs based on binarized data (Shmulevich et al., 2002; Hopfensitz et al., 2012) as in this work. Additionally, BASC algorithms handle the presence of noise (interferences in the gene expression measurement process) and the presence of few measurement points (as it can be costly to obtain them) (Ferreira, 2019). For this work, values above the threshold are set to 1 (active), and values below are set to 0 (inactive) (Weaver et al., 1999; Di Cara et al., 2007).

### 2.5 Trajectory simulation

The trajectory simulation was performed by ASSA-PBN in a bash script (see supplementary data repository), set to execute the simulation with n steps. The result is a TXT file containing the trajectory.

A trajectory shows the sequence of states in the evolution of a network, i.e., how state transitions occur, for analysis of its dynamic behavior. For this simulation, the initial states of each node need to be established as input parameters. Due to the size of the core sub-network (212 nodes), there is an enormous number of possible state vectors, making it infeasible to simulate them starting from each node or choosing them randomly (Ferreira, 2019). These states must make biological sense, so the binarized RNA-seq expression data define the initial states.

Using ASSA-PBN, trajectory simulations were performed with 30,000 state transitions based on the binarized RNA-seq data.

With the rules determining the model and the binarized RNA-seq values assigned to each node in the defined core sub-network, its stable equilibrium states, i.e., the attractors, which can be single (composed of a single state) or cyclic (composed of multiple states), are explored (de León et al., 2022). As explained above, the basins of attraction are all the states of the system that evolve toward a specific attractor, and these states represent the epigenetic barriers that delimit the basin of attraction (Conforte et al., 2020). To illustrate this, The basins corresponding to the bulk RNA-seq dataset were obtained using another Python script developed by our group. The trajectory file generated by ASSA-PBN is the input for this algorithm, which analyzes the conserved genes in the last n time steps.

Considering that all obtained basins have cyclic attractors, the expression behavior of all specific genes in the core sub-network was evaluated at each time interval, observing whether they varied in their Boolean value during the attractor cycle or remained fixed for the entire cycle. The script selects only the genes that did not show variations in Boolean values in any of the attractors over the last n time steps of the simulation. In this work, *n* = 1000, meaning that out of the 30,000 steps of the trajectory simulation, the script selected genes whose binary values remained unchanged in the last 1000 steps. These constant genes constitute the basin of attraction. The script generates an output file as a table listing the genes in the basin and their respective bar plot.

## 3 Results

### 3.1 Boolean model of the *P. aeruginosa* CCBH4851 GRN

More information about the core sub-network Boolean model is described in Table 1. It is important to emphasize that the network described in Table 1 is a sub-network of CCBH-2022 that dictates its dynamics. One first observation is that the core network is much smaller than the CCBH-2022 GRN. Another fact worth noting is that most nodes are associated with just one Boolean function. Most regulatory interactions are positive and are related to gene activation, as shown in Table 1.

**TABLE 1:** Description of the Boolean model of CCBH-2022.

Sub-network nodes (total)	212
Nodes with incoming and outgoing edges	166
Nodes with outgoing edges	46
Sub-network edges (total)	504
Positive regulation	239
Negative regulation	124
Dual regulation	3
Unknown regulation	138
Autoregulation (total)	89
Positive autoregulation	30
Negative autoregulation	40
Unknown autoregulation	19
Number of nodes in Boolean model	212
Number of nodes with 1 function	154
Number of nodes with 2 functions (chance of 50% for each)	58
Number of functions in Boolean model (total)	270
Number of positive autoregulation functions	57

### 3.2 The proposed method for identifying biologically relevant basins of attraction is computationally efficient

**FIGURE 3:**
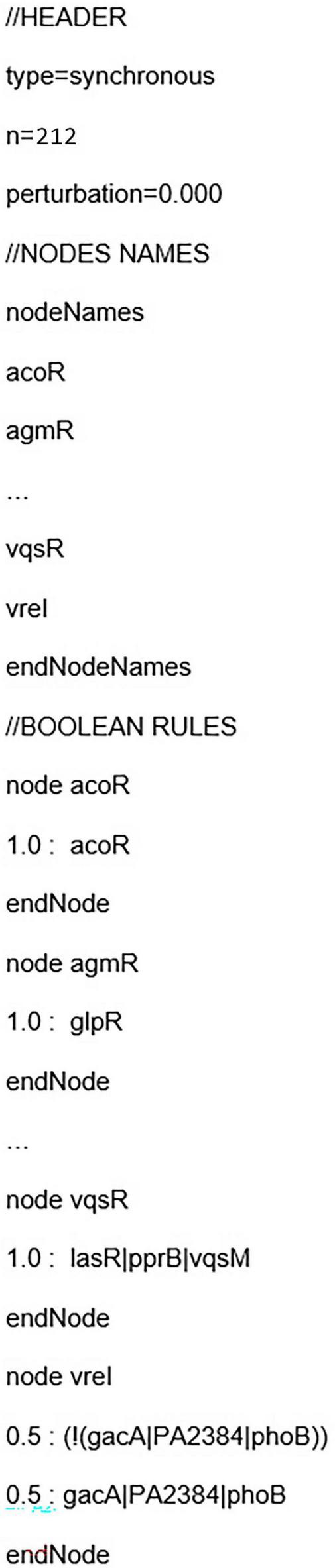
Abstract of the “.pbn” file. The summarized model code in “.pbn” format of the simplified network (core sub-network) of CCBH-2022. The “.pbn” file includes the simulation type (type), the number of network nodes (n), a perturbation factor (perturbation), a list of network node names (nodeNames), and the definition of Boolean functions for each node, accompanied by the respective probabilities for nodes that are not completely determined (typically set at 50% for functions expressed and unexpressed in double interactions or unknown cases).

The model in the “.pbn” format (for more information, please see Mizera et al., 2018) of the simplified network (core sub-network with 212 genes) of CCBH-2022 is summarized in Figure 3, and the complete code is available in the supplementary data repository. It includes the simulation type (type), the number of nodes in the network (n), a perturbation factor (perturbation), the list of network nodes (nodeNames), and the definition of the Boolean functions for each node, preceded by the respective probabilities of the partially defined nodes (in this case, 50% for expressed and unexpressed functions in double or unknown interactions) (Shmulevich et al., 2002). According to Xiao and Dougherty (2007), perturbation in the case of a Boolean network, which is a rule-based binary network, refers to how a small change in a regulatory rule, such as inverting its original binary value, affects the steady state of the network. ASSA-PBN allows the application of a perturbation value to nodes in the Boolean model. For example, if a node with a constant Boolean value, contributing to a basin of attraction, has its state perturbed, it may start varying and leave that basin. For this reason, the perturbation factor in this work is set to 0, meaning no perturbation is allowed.

**FIGURE 4:**
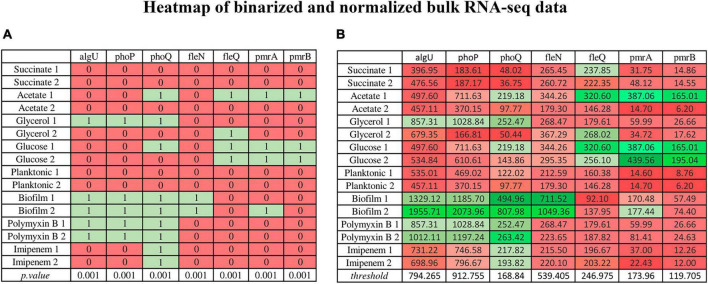
Heatmap illustrating binarized bulk RNA-seq data for selected genes (*p*-value > 0.001) alongside their normalized data (Transcripts Per Million) on a gradient color scale. **(A)** The binarized data heatmap (*p*. value > 0.001), in which green represents activation, red indicates inhibition. **(B)** The normalized data heatmap, in which the color scale reflects threshold values, where stronger shades of green indicate values above the threshold, and deeper red hues signify values below it. The normalized gene heatmap showcases behavior based on the threshold identified by BASC-B.

Figure 4 is an example of binarized bulk RNA-seq data for the selected genes discussed in this work, represented as a heatmap (*p*. value > 0.001) (Figure 4A), also displaying their corresponding normalized data (Figure 4B) (Transcripts Per Million) on a gradient color scale. Their activation (depicted in green) or inhibition (in red) in each RNA-seq condition in both scenarios can be seen. In the heatmap of normalized genes, their behavior according to the threshold detected by BASC-B is observed.

**FIGURE 5:**
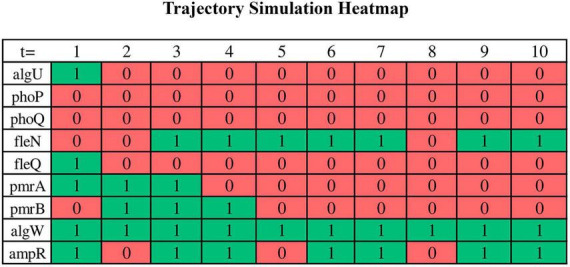
A portion of the trajectory simulation output by a bash script for some of the nodes in a heatmap. Binarized RNA-seq data serves as the starting points for the trajectory, and the results of the trajectory simulation are examined in the form of a “.txt” file. However, a segment of this data has been adapted and presented in this heatmap to enhance visual interpretation. Each row corresponds to the Boolean state values of the respective genes over the course of the trajectory (green for activation and red for inhibition), while the columns represent the various time steps during the simulation. In this simulation, ASSA-PBN is instructed to carry out simulations encompassing 30,000 state transitions. The specific command lines are detailed in the supplementary data repository.

Trajectory simulations of all genes in the core sub-network for the 16 samples were performed using the GRN_to_ASSA.py script (supplementary data repository). It took approximately 1 min on an Ubuntu notebook (22.04 LTS) with an Intel Core i5 (2.5 GHz) processor, 8 GB of RAM, and 1 TB HD, demonstrating computational efficiency to run a single simulation. We run a total of 16 trajectory simulations, one for each replicate. Additional executions per replicate provided the same result as the first run when considering the set of genes that characterize a basin of attraction, which is a consequence of choosing a large number of steps for trajectory simulation. The binarized RNA-seq data were used as the initial points of the trajectory. The trajectories are expected to converge to simple or complex attractors within basins of attraction corresponding to phenotypes. The result is a “.txt” file containing all gene states for each time step of the trajectory, which can be read in Excel. To enhance visual representation, the initial section of the file (first 10 steps in the trajectory) has been adapted into a heatmap graph for select genes in Figure 5. This heatmap is from a biofilm sample (experiment #12). Each row in the graph corresponds to the Boolean value of the states of individual genes at each step of time in the trajectory (green: activation/1; red: inactivation/0), while the columns represent the different time steps in the simulation.

In addition to the CSV file with the dynamics during the 30,000 steps of all 212 genes (in heatmap format in Figure 5), the GRN_to_ASSA.py script also generates the “conserved_genes.csv” (in the supplementary data): a separate table with the 46 genes that only have output edges.

### 3.3 Every condition is associated with different regions of the epigenetic landscape

**FIGURE 6:**
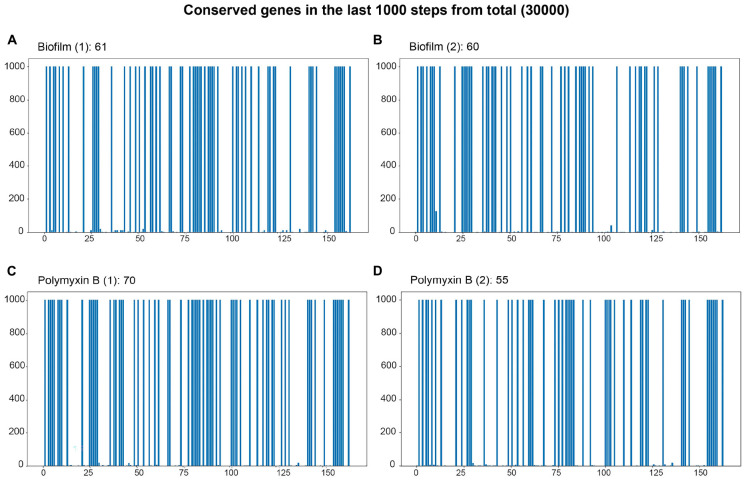
Bar plot of the final 1000 steps out of 30000 simulation steps. The *x*-axis represents the genes present in the simulation, and the *y*-axis represents the last 1000 time steps of the trajectory. Genes that maintain a filled blue bar extending to the top of the *y*-axis are the genes whose states are stable and define the attractor basin of the trajectory. **(A,B)** Genes characterizing the attractor basins of both biofilm samples. **(C,D)** Genes defining the basins of attraction for the two polymyxin B samples.

For the detection of basins of attraction, the find_basin.01.py script (supplementary data) reads the dynamics file (heatmap in Figure 5) and the “conserved_genes.csv” file. The algorithm considers the 46 genes based on the dynamics of the Boolean model but separates them in the final result, excluding them from the bar plots (Figure 6) and their respective tables (output files of the algorithm). These 46 genes will always be part of the basins of attraction for all experiments since their states remain fixed throughout the trajectory simulation. Their binary values are obtained from the simulated RNA-seq sample. They remain constant in all samples because it is through the input edge that the state of a gene can be modified. Therefore, the final result includes 166 genes with input and output edges whose stable states we want to analyze.

**FIGURE 7:**
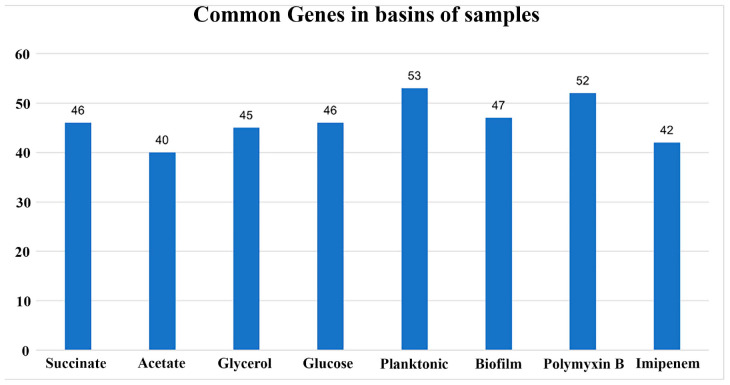
Barplot of the number of common genes in the basins of the samples and their duplicates. The RNA-seq experiments were conducted in duplicates for each condition. The graph illustrates the number of common genes between sample duplicates for each RNA-seq condition used in this study. The *x*-axis represents the conditions, and the *y*-axis represents the number of shared genes between each pair of samples.

Out of the 166 genes, the genes with constant states in the last 1000 steps (total steps: 30,000) of the simulations for all 8 conditions of the 16 RNA-seq experiments were determined. The number of shared genes between the basins of the samples and their respective duplicates is presented in Figure 7. Every condition is characterized by a different gene set, which indicates that every condition is associated with a different region of the epigenetic landscape. Nevertheless, it is important to highlight that more than one basin of attraction may be present in the region of the epigenetic landscape related to a condition.

### 3.4 Phenotypically relevant genes associated with *biofilm formation* and *polymyxin sensitivity* basins of attraction

For the association of basins with phenotypes, we chose two conditions out of the eight that have substantial data in the scientific literature: biofilm and polymyxin B. The bar plots for the biofilm and polymyxin B simulations are shown in Figure 6. The blue columns up to 1000 on the *y*-axis represent genes with constant values in the last 1000 steps of the 30,000-step trajectory simulation. They are all in the same basin of attraction. It is important to note that the gene values can be either 0 or 1; what is relevant is that this value remains constant in the last 1000 steps.

Figures 6A, B correspond to the basins of the 2 biofilm phenotype experiment samples. Basin A contains 61 genes with constant Boolean values, while Basin B contains 60. Figures 6C, D 3 represent the samples under the polymyxin B condition, with C having 70 genes in the basin and D having 55. It is important to mention that more than one basin may be associated with a condition, representing different local minima within the same basin of attraction. For instance, the epigenetic region associated with biofilm is identified by the conserved genes in both basins A and B. Due to its size, the list of genes contained in the attraction basins of the Biofilm and Polymyxin B samples is available in “biof-polb-basin.xlsx” within sim.zip in the supplementary data repository. The analysis of the dynamic behavior of the network can be assessed through trajectory analyses, aiming to identify the most frequent transitions. From these transitions, it is possible to find genes whose state changes had the most impact on these transitions through phenotype analyses.

Figure 6 shows several genes with a constant Boolean value in CCBH-2022 at various trajectory steps during simulation. This stable set of states can be detected by the cyclic repetition of Boolean values in the network genes, with these genes being related to the stability of the attractor. The essential genes in the basins were identified, and some were associated with phenotypes through literature research for this work. This allows us to formulate hypotheses about the attractors. As described in the section “2. Materials and methods,” two phenotypes were selected to evaluate the validation of the Boolean model: biofilm and polymyxin B, based on the availability of more information in the scientific literature.

**FIGURE 8:**
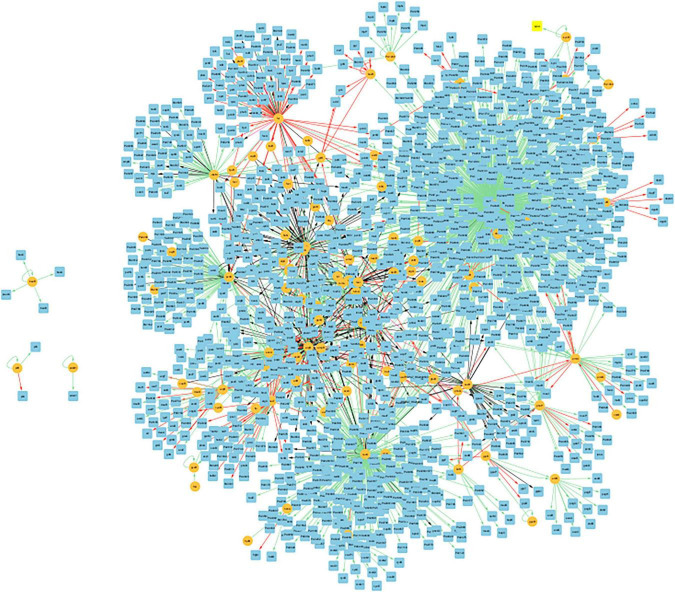
Visualization of the GRN of all gene interactions of the 47 common genes present in the biofilm basin of attractions within the CCBH-2022. Illustration of the gene interactions between the genes found in the biofilm attractor basin and the rest of the CCBH-2022 network. Yellow circles indicate regulatory genes, light blue circles indicate target genes, black lines indicate an unknown mode of regulation, green lines indicate activation and red lines indicate repression. Purple lines indicate a dual-mode of regulation. It is possible to observe the larger, highly interconnected component, along with three regulatory genes and their targets that are not connected to the larger component. The image was created using Cytoscape (version: 3.9.1).

Figure 8 presents the biofilm GRN of all gene interactions for genes present in the biofilm basin of attraction. It has 2162 edges, and these interactions were classified into activation (“ + “), repression (“-”), dual (“d,” indicating that the regulatory gene can function as both an activator and a repressor depending on the conditions), and unknown (“?”), described in dedicated biological databases and scientific literature. The image was created using Cytoscape (version: 3.9.1).

## 4 Discussion

Certain genes associated with the phenotypes in the RNA-seq conditions are indeed present in the CCBH-2022, but solely as targets, characterized by having only incoming edges, without any regulatory role. Consequently, they are not part of the essential core sub-network and, consequently, and, therefore, are not present in the basin of attraction of any condition. This include, for example, genes related to glucose metabolism in *P. aeruginosa*, such as *glk* (Glucokinase), *gapA* (Glyceraldehyde-3-phosphate dehydrogenase), *edd* (6-Phosphogluconate dehydratase), and acetate-related genes like *acsA* (Acetyl-CoA synthetase) and *ackA* (Acetate kinase). Consequently, their activation or inhibition states may undergo alterations, but they do not impact other network nodes. Although they are integrated within the GRN, they function as regulated elements without contributing to the characterization of the basin of attraction.

There were 61 genes in the basin of attraction for the first biofilm sample and 60 genes in the second sample. Among the two basins, there were 47 genes in common. These 47 genes define the larger basin of attraction, while the number of possible states for the 60 genes basin is smaller and nested within the larger basin. In the polymyxin B samples, the first sample had 70 genes, and the second had 55. The difference in the case of biofilm lies in the presence of the *czcR* gene in one sample of each condition. This gene modulates the repression of pyocyanin production and biofilm formation in the presence of an excess of Zn^2+^ or ZnO nanoparticles (Lee et al., 2014), in addition to modulating quorum sensing and antibiotic resistance through direct binding with gene promoters such as *lasI*, *phzA1*, and *oprD* (Dieppois et al., 2012). It is also essential for flagellar gene expression and swimming motility during Zn^2+^-induced stress (Liu et al., 2022). The association of multiple basins of attraction with biofilms is compatible with the physiological variation of bacteria growing in biofilms, as reported previously for *P. aeruginosa* (Williamson et al., 2012).

Figure 8 displays a GRN containing only the genes present in the biofilm basin of attraction and their gene interactions with the rest of the CCBH-2022. This biofilm GRN includes the 47 genes common to both biofilm samples, which belong to the largest attractor basin. Interactions were considered in which these genes function as both regulators and targets. As a result, these 47 nodes are involved in 2162 gene interactions, acting as both regulators and targets. This Figure shows that this biofilm GRN is highly interconnected, with only three regulatory genes and their targets disconnected from the larger component. According to Chagas et al. (2022), this variability in the number of connected components is most likely linked to the availability of biological information used for the interaction reconstruction. This illustrates how biofilm formation is a complex phenomenon emerging from the interactions of numerous genes.

The biofilm phenotype can be described in terms of genes expressed by cells associated with the biofilm (Donlan and Costerton, 2002). Biofilms are microbial communities of cells attached to a substrate, an interface, or to each other and embedded in a matrix of extracellular polymeric substances produced by them (Donlan and Costerton, 2002).

Long-term infections, such as those in the lungs of cystic fibrosis patients, are maintained through the conversion of *P. aeruginosa* to a mucoid phenotype, which results from the overproduction of the exopolysaccharide alginate (Blanco-Cabra et al., 2020). Biofilm formation is a complex and multifactorial process, but studies have shown that this overproduction leads to highly structured biofilms and is responsible for some properties of the biofilm, such as increased resistance to the antibiotic tobramycin (Sans-Serramitjana et al., 2017). Biofilms formed by mucoid *P. aeruginosa* contain significant amounts of alginate, which influences the architecture of the biofilm (Govan and Deretic, 1996). In non-mucoid strains such as PAO1, alginate is not the predominant polysaccharide present in non-mucoid biofilms of *P. aeruginosa* cultured *in vitro*, and it is not necessary for biofilm development (Stapper et al., 2004), likely being a polysaccharide produced under stress (Hentzer et al., 2001). The conversion of non-mucoid *P. aeruginosa* to the mucoid phenotype, characterized by the overproduction of alginate, is a critical step in the pathogenesis of cystic fibrosis, and it worsens the prognosis for the patient (Hentzer et al., 2001). Therefore, activating the alginate biosynthetic pathway, and developing biofilms in *P. aeruginosa*, represent a critical moment in cystic fibrosis pathology (Morici et al., 2007). The conversion to mucoid phenotype occurs, in most cases, through spontaneous mutations in *mucA*, leading to the hyperactivity of *algU*, which in turn results in the overexpression of the alginate operon (Potvin et al., 2008). Therefore, it plays a key role in forming mucoid biofilms in *P. aeruginosa*. Bazire et al. (2010) examined the potential role of *algU* in forming non-mucoid biofilms in *P. aeruginosa* and found that *algU* is critical for forming robust biofilms. The *algU* gene is present in the biofilm basin with a value of 1, indicating that it is always active in this basin.

The two-component regulatory system consists of a transmembrane kinase sensor and its respective regulator and is responsible for mediating bacterial responses to various environmental stimuli (Groisman, 2016). Wu et al. (2021) showed that when the expression levels of the PhoP-PhoQ two-component system were disrupted, there was a reduction in biofilm formation and cellular motility in *P. aeruginosa* PAO1 △phoP or △phoQ mutants. These genes are essential for bacterial virulence and regulate gene expression in quorum sensing, flagellar assembly, and biofilm formation (Gooderham et al., 2009). Both *phoP* and *phoQ* are present in the biofilm basin with a value of 1 (active).

*fleN* plays an important role in regulating flagellar gene expression and flagellar biogenesis and in the transcriptional regulation of biofilm matrix components (Navarrete et al., 2019). This gene is present with a value of 1 in the biofilm basin. On the other hand, *fleQ*, in addition to being a master regulator of flagellar gene expression, also derepresses the expression of genes involved in biofilm formation when intracellular levels of cyclic guanosine monophosphate (cGMP), a nucleotide acting as a second messenger, are elevated (Varadarajan et al., 2020). Unlike flagellar genes, genes associated with biofilms are not always easily recognizable in genome sequences (Gilleland and Murray, 1976). Baraquet and Harwood (2015) identified new genes regulated by *fleQ* involved in biofilm formation. FleQ is part of the basin of attraction with a value of 0 (inactive). Navarrete et al. (2019) revealed that FleN acts synergistically with FleQ in the activation of transcription of important genes involved in biofilm formation and *in vivo* cell-surface and cell-cell interactions at high levels of cyclic di-GMP (c-di-GMP) but limits FleQ-dependent activation under low c-di-GMP conditions. This may be a possible reason for the activation of one and the inactivation of the other in biofilm samples.

Since CCBH4851 is sensitive to polymyxin B, we focused on genes related to its resistance. The phenotype of adaptive polymyxin B resistance was first reported by Gilleland and Murray in 1976 using the wild-type PAO1 strain in a low Mg^2+^ medium and exposed to increasing concentrations of polymyxin B. Since then, many studies have focused on the structural basis of adaptive resistance (Santajit and Indrawattana, 2016), and it has been discovered that under various environmental conditions, *P. aeruginosa* synthesizes different forms of the lipid portion (lipid A) of lipopolysaccharide (Gilleland and Farley, 1982), particularly under Mg^2+^-limiting conditions.

In *P. aeruginosa*, the acquisition of polymyxin resistance is mainly due to mutations in two two-component regulatory systems, PhoP-PhoQ and PmrA-PmrB, which respond to Mg^2+^-limiting conditions, resulting in polymyxin B resistance (Schurek et al., 2009), and self-regulating operons (Gilleland and Conrad, 1982).

The PhoP-PhoQ system is associated with bacterial resistance to polymyxins (Yang et al., 2021) and multidrug efflux pumps. Yang et al. (2021) revealed that PhoP-PhoQ contributes to bacterial tolerance to polymyxin B by directly regulating numerous genes involved in LPS modification and membrane integrity maintenance. These genes may also be involved in the bacterial response to environmental stresses such as cation depletion and antimicrobial substances from the host. Both *phoP* and *phoQ* are part of the basin of attraction with a value of 1, indicating that they are active.

On the other hand, the PmrAB system can promote bacterial resistance to polymyxins and cationic peptides in response to Mg^2+^ deficiency by modifying lipopolysaccharide molecules (Schurek et al., 2009). Xiao et al. (2022) showed that all polymyxin-resistant isolates evaluated in their study had mutations in *pmrB*. Both *pmrA* and *pmrB* are present in the basin of attraction, but they are inactive, possibly explaining why the CCBH4851 strain is still sensitive to polymyxin B.

The method described here can be applied to any other organism as long as similar data is available and organized to be used following the procedures described in section “2. Materials and methods.”

Due to the size of CCBH-2022, a simplification approach was used to focus on the core sub-network resulting from modeling its dynamics to reach steady states within the basin of attraction. This simplification involved literature research in separating genes with input and output edges as criteria for selecting the components of the core sub-network.

Since cellular regulation is strongly linked to gene expression, information related to gene expression was used as input data to simulate the model’s dynamics. In this work, we used recently obtained bulk RNA-seq data from our group, which underwent a normalization process and were subsequently binarized for the Boolean model treatment.

The interactions identified in CCBH-2022 were used to infer the Boolean functions of each node in the core sub-network. The nature of CCBH-2022’s gene regulatory network, which contains both dual and unknown interactions, gave the model characteristics of probabilistic Boolean networks, and a 50% chance of choosing either the activation or inhibition Boolean function was adopted (Shmulevich et al., 2002). The stochastic nature of the model has a direct implication in the complexity of the observed attractors. On determinist models, one expects that most attractors are composed of a single state or a limited set of state nodes in a cycle. In particular situations, chaotic attractors can emerge in Boolean networks (Karlsson and Hörnquist, 2007). For the PBN described in this manuscript, it is possible to note that most complex attractors do not compose a closed cycle but behave more like chaotic attractors due to the stochastic nature of the model (Schwab et al., 2020). That justifies our choice to identify those genes that remain constant over a large part of the trajectory to characterize basins of attraction.

Multiple trajectory simulations were performed on this core sub-network to analyze the evolution of the network and identify basins of attraction where genes remained in stable states. The analysis of the dynamic behavior of the model revealed characteristics of a gene regulatory network, such as robustness and a tendency to return to a stable state.

Overall, this work provides new insights into identifying new antibiotic targets and contributes to an increased understanding of the behavior of this bacterium. Two scenarios are possible: either a potential target can be inhibited (Kerr et al., 2022) or be a subject of a gene knockout (Dallidis and Karafyllidis, 2014). Both scenarios are related to perturbations in the current state of the system. The first scenario implies a perturbation in a trajectory that may result in a change of basin of attraction. The second scenario may cause a structural change in the core sub-network. In either case, the set of genes that remain constant for a given condition indicates potential candidates for intervention. In the context of Boolean modeling (Trunk et al., 2010), perturbations can be applied to gene interactions, altering the logical rules or connections between genes. Perturbations can lead to changes in system behavior, such as activating or inhibiting specific pathways. Perturbations can also be applied to gene expression, increasing or decreasing the expression of one or more genes by changing the logical state of a gene from “active” to “inactive” or vice versa. Perturbations in the initial conditions of the Boolean model, which represent the initial state of genes in the trajectory simulation and may be associated with variables external to the cell, can also be explored. Perturbations can help us investigate how the system responds to changes and deviations from the normal state and which perturbations may influence the behavior of genes and their interactions by observing the system’s dynamics. Therefore, it will be a valuable analysis to understand gene regulation mechanisms better, identify essential signaling pathways, and try to predict the effect of perturbations on complex biological systems (Xiao and Dougherty, 2007).

## 5 Conclusion and perspectives

In this report, the Boolean model and complex dynamics of the regulatory gene system of *P. aeruginosa* CCBH4851 were modeled using a PBN paradigm, and RNA-seq bulk data were used to identify basins of attraction. The methodology presented here can be extended to any organism with similar data and can be directly applied to analyze other bacterial species, such as *Escherichia coli*. This work can be expanded in various ways, such as designing an algorithm to define therapeutic interventions based on model analysis. The idea is to manipulate the model toward desirable states through targeted intervention in specific genes. This systems biology approach could lead to developing strategies to disrupt the connectivity of these essential processes within the basins of attraction, potentially reducing the pathogenicity and suppressing the resistance of this bacterium.

The results of this research will be used for integration with the metabolic network of *P. aeruginosa* CCBH4851, which is currently under development by our research group. Several integration methods are described in the literature (e.g., Covert et al., 2008; Motamedian et al., 2017). We also intend to incorporate other cellular processes into the integrated model, advancing toward a whole-cell model (Ahn-Horst et al., 2022) of a multidrug.resistant *P. aeruginosa*. Additionally, future analyses should include perturbations in the Boolean modeling of CCBH-2022 to evaluate changes in its dynamics and whether they align with the available literature.

Additionally, data integration and drug repositioning analyses will be explored to develop new treatment strategies for infections caused by this bacterium and contribute to identifying new antibiotic targets. This case involves combining genomic and pharmacological information to identify existing drugs that can be repurposed for treating diseases different from those developed initially (Badkas et al., 2021). This process helps accelerate the discovery of new uses for existing drugs by leveraging knowledge about gene interactions and signaling pathways involved in the disease. Boolean modeling of biological networks provides a simplified representation of these interactions, enabling efficient computational analysis and the identification of promising candidates for further experimental validation (Dallidis and Karafyllidis, 2014; Recanatini and Cabrelle, 2020; Kerr et al., 2022).

## Data availability statement

The original contributions presented in the study are publicly available. This data can be found here: https://github.com/marciachagas/CCBH4851-boolean-model.

## Author contributions

MSC: Analysis, Investigation, Data curation, Writing – original draft. MTS: Methodology, Software, Writing – review and editing. MAM: Writing – review and editing. FABS: Writing – review and editing, Conceptualization, Funding acquisition, Investigation, Methodology, Supervision.
